# Development of a porcine model of phenylketonuria with a humanized R408W mutation for gene editing

**DOI:** 10.1371/journal.pone.0245831

**Published:** 2021-01-25

**Authors:** Robert A. Kaiser, Daniel F. Carlson, Kari L. Allen, Dennis A. Webster, Caitlin J. VanLith, Clara T. Nicolas, Lori G. Hillin, Yue Yu, Catherine W. Kaiser, William R. Wahoff, Raymond D. Hickey, Adrienne L. Watson, Shelley R. Winn, Beat Thöny, Douglas R. Kern, Cary O. Harding, Joseph B. Lillegard

**Affiliations:** 1 Department of Surgery, Mayo Clinic, Rochester, Minnesota, United States of America; 2 Midwest Fetal Care Center, Children’s Hospitals and Clinics of Minnesota, Minneapolis, Minnesota, United States of America; 3 Recombinetics, Inc., St. Paul, Minnesota, United States of America; 4 Faculty of Medicine, University of Barcelona, Barcelona, Spain; 5 Department of Molecular Medicine, Mayo Clinic, Rochester, Minnesota, United States of America; 6 Department of Molecular and Medical Genetics, Oregon Health & Science University, Portland, Oregon, United States of America; 7 Department of Pediatrics, University of Zurich, Zurich, Switzerland; 8 Pediatric Surgical Associates, Minneapolis, Minnesota, United States of America; Nathan S Kline Institute, UNITED STATES

## Abstract

Phenylketonuria (PKU) is a metabolic disorder whereby phenylalanine metabolism is deficient due to allelic variations in the gene for phenylalanine hydroxylase (*PAH*). There is no cure for PKU other than orthotopic liver transplantation, and the standard of care for patients is limited to dietary restrictions and key amino acid supplementation. Therefore, *Pah* was edited in pig fibroblasts for the generation of PKU clone piglets that harbor a common and severe human mutation, R408W. Additionally, the proximal region to the mutation was further humanized by introducing 5 single nucleotide polymorphisms (SNPs) to allow for development of gene editing machinery that could be translated directly from the pig model to human PKU patients that harbor at least one classic R408W allele. Resulting piglets were hypopigmented (a single Ossabaw piglet) and had low birthweight (all piglets). The piglets had similar levels of PAH expression, but no detectable enzymatic activity, consistent with the human phenotype. The piglets were fragile and required extensive neonatal care to prevent failure to thrive and early demise. Phenylalanine levels rose sharply when dietary Phe was unrestricted but could be rapidly reduced with a low Phe diet. Fibroblasts isolated from R408W piglets show susceptibility to correction using CRISPR or TALEN, with subsequent homology-directed recombination to correct *Pah*. This pig model of PKU provides a powerful new tool for development of all classes of therapeutic candidates to treat or cure PKU, as well as unique value for proof-of-concept studies for *in vivo* human gene editing platforms in the context of this humanized PKU allele.

## Introduction

Phenylketonuria (PKU; OMIM 262600) is one of the most common inborn errors of metabolism of the liver, affecting approximately 1 in every 10,000–14,000 live births [1, 2]. Typically, PKU is the result of genetic mutations causing deficiencies in phenylalanine hydroxylase (PAH) activity, which catalyzes the conversion of phenylalanine to tyrosine [3]. Untreated, PKU causes severe behavioral problems and neurocognitive impairment [4]. Contemporary therapy involves dietary phenylalanine restriction for all patients, along with oral administration of tetrahydrobiopterin cofactor for patients with partial-activity *PAH* alleles [5]. Enzyme substitution therapy or orthotopic liver transplantation are expensive options with lifelong implications. However, there is currently no true cure for PKU short of liver transplantation.

PKU is recessive, and many patients are compound heterozygous for two different variants at the *PAH* locus, demonstrating a spectrum of severity resulting from partial to complete loss of PAH activity [6]. There is a high degree of variation between PAH activity levels based on the exact *PAH* alleles present [7]. Although PKU is phenotypically complex [4] most patients typically assume a metabolic phenotype dictated by the less-severe allele. Functional correction of the mutant *PAH* gene, or transgene expression of wild type *PAH* in a sufficient number of hepatocytes could cure PKU for many patients [8], and correction of only one allele is required to cure any specific hepatocyte. The number of corrected hepatocytes that would be required to cure a patient is estimated to be at least 10%, based on severity of alleles present [9, 10]. This relatively low target makes PKU an interesting indication for gene therapy, but there are still many challenges to developing these advanced approaches [11].

PKU researchers have benefitted by the availability of useful mouse models for decades [12], but there were no clinically-relevant large-animal models. Therefore, we chose to create an R408W pig model of PKU, selecting this common and severe disease allele that results in complete ablation of PAH activity [7, 13, 14] and is present in 40% [15] to 70% [14, 16] of patients depending on nationality. In creating this model, we humanized 5 SNPs around the R408W locus (hR408W), as this genotype is often inherited with these SNPs [13]. Such a pig should be 1) a relevant model of the human disease arising from null-activity PAH expression, 2) genetically consistent to breed or clone due to lack of heterogeneity at this locus, and 3) definitive in the preclinical evaluation of any potential therapeutic approaches for PKU.

Developmental studies in a pig model with any two generally-null alleles could be translated directly to patients for non allele-specific treatments of PKU, such as oral administration of supplements, probiotics, dietary control of circulating phenylalanine, and gene delivery of *PAH* or phenylalanine ammonia-lyase (*PAL*). However, differences in the target sequence that would be apparent in varied null alleles, or those species-specific differences between model and human could render many investigational gene editing reagents for humans ineffective in a pig model. By electing to not only create the pathogenic human amino acid substitution R408W in these pigs, but also to humanize the nearby polymorphisms, the *Pah*^hR408W/hR408W^ pig has 48 contiguous base pairs that fully represent the human disease allele (hR408W). CRISPRs, TALENs, prime editor, or other gene therapy platforms that rely on sequence identity within target regions could be developed in this model, and results would be directly translatable to human patients harboring at least one R408W allele without the need to develop expensive species-specific models to test safety or efficacy.

Here we show the development of this novel *Pah*^hR408W/hR408W^ PKU pig model, including phenotypic characterization. We anticipate that this porcine PKU model will expedite development of advanced therapies for PKU and could also serve as a platform for demonstrating general safety and utility of other exploratory gene editing platforms in the context of a human allelic target.

## Results and discussion

**Fig 1:**
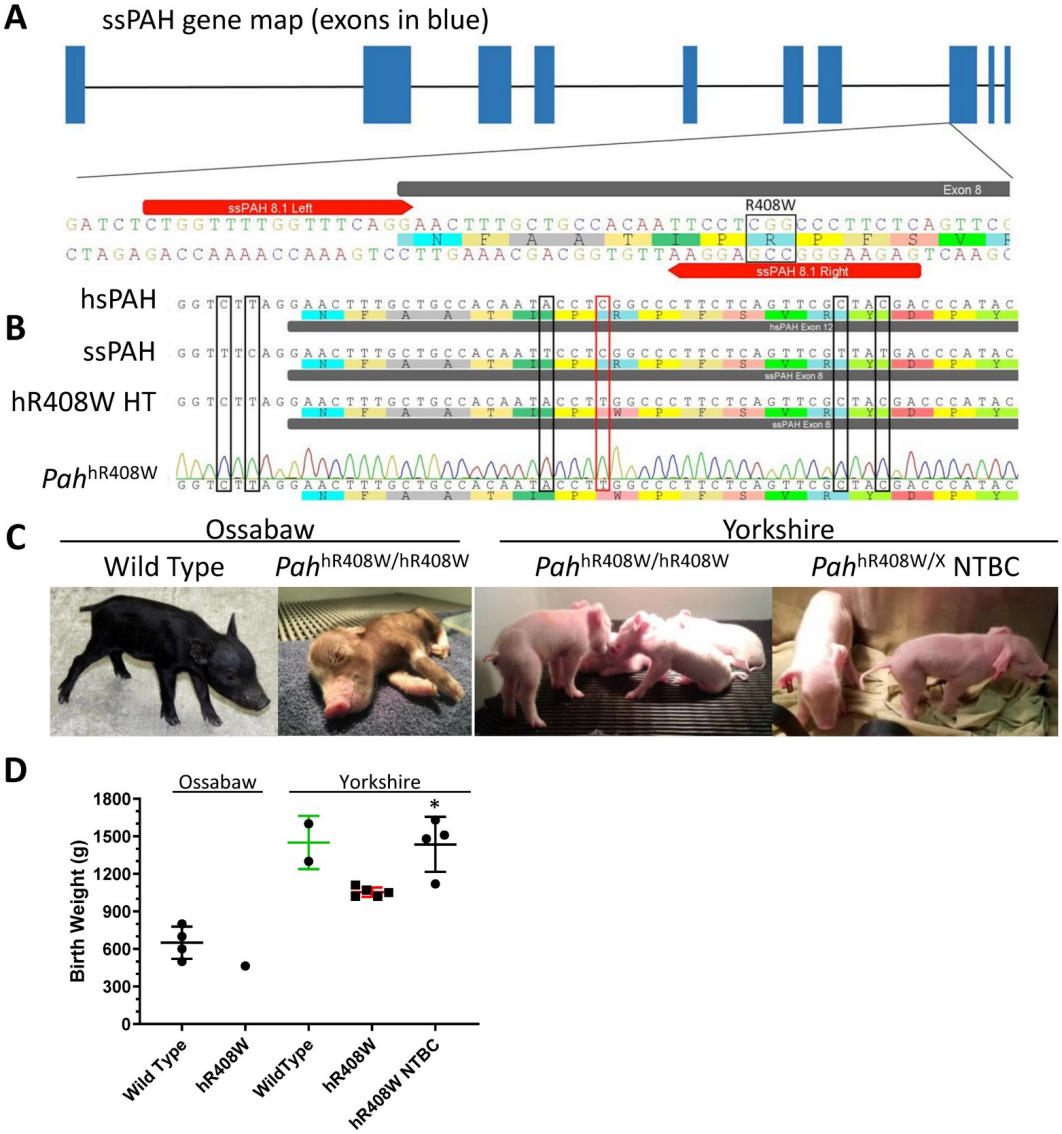
Production of *PAH*-targeted piglets. A) TALENs were designed to target Exon 8 of sus scrofa (ss) *PAH*. The location of the right monomer was strategically placed to contain a mismatch following a successful R408W HDR event. B) Sequence alignment of human (hs, top) and wild type pig (ss, second) shows the high similarity surrounding the target R408W mutation (red box). A homology template (HT, third) was designed to produce the R408W mutation in addition to introducing the 5 SNPs (black boxes) needed to “humanize” the sequence (hR408W) around the mutation to allow for targeting with human-translatable gene editing reagents. Lastly, genotype confirmation verified all piglets were positive for R408W and the 5 humanizing SNPs. C) Picture of wild type Ossabaw piglet (left) and Ossabaw *PAH*^hR408W/hR408W^ founder piglet (middle left, No. 1769) showing hypopigmentation with hR408W mutation consistent with that observed in PKU mouse models. Additionally, large white/landrace PKU founders (*PAH*^hR408W/hR408W^, middle right) and PKU founders gestated on NTBC (*PAH*^hR408W/A403GfsX47^, far right) are also presented. D) Birth weights of these *PAH*-targeted piglets are lower than historical values for wildtype piglets of their respective genetic background strains in the absence of NTBC during gestation. * p < 0.05 compared to untreated *PAH*^hR408W/hR408W^ in the same background.

To create the model, we used TALENs targeted to exon 8 of the *PAH* allele in pig fibroblasts (Fig 1a) and a single stranded oligodeoxynucleotide (ssODN) homology template to include the R408W mutation as well as incorporate the 5 human SNPs (Fig 1b). Early passage fetal fibroblasts from an Ossabaw minipig or Yorkshire (large-white conventional pigs) were transfected, subcloned, and screened by Sanger sequencing to identify clones that harbored the desired allele. Positive clones were pooled and used as donor cells for somatic cell nuclear transfer (SCNT) to create founder piglets. Five total pregnancies resulted in ten live born piglets. Since these animals were cloned (not bred) they were all homozygous *PAH*-targeted (PKU) animals, all harboring at least one hR408W allele (Table 1). An eleventh piglet died of complications during the delivery procedures unrelated to the PKU phenotype. No effects of *Pah* targeting were evident on litter size, as the total number of offspring was consistent with historical averages for cloned piglets [17].

**Table 1:** Genotypes of all *PAH*-targeted piglets.

Pig ID	Breed	Lifespan	Genotype
Pregnancy A			
1769	Ossabaw	4 days	hR408W Homozygote
Pregnancy B			
1794	Yorkshire	5 days	hR408W Homozygote
1795	Yorkshire	2 days	hR408W Homozygote
1796	Yorkshire	2 days	hR408W Homozygote
1797	Yorkshire	3 days	hR408W Homozygote
Pregnancy C			
1798	Yorkshire	5 days	hR408W Homozygote
Pregnancy D (NTBC)			
21 (still born)	Yorkshire	0 days	hR408W Homozygote
22 Charm	Yorkshire	6 days	Compound Heterozygote—hR408W; pA403GfsX47^1^
23 Lucky	Yorkshire	200 days^§^	Compound Heterozygote—hR408W; pA403GfsX47^1^
Pregnancy E (NTBC)			
899 Cornflake	Yorkshire	96 days	hR408W/R408W compound heterozygote- one allele lacks the two 5’ humanized bases^2^
900 Cheerio	Yorkshire	2 days	hR408W/R408W compound heterozygote- one allele lacks the two 5’ humanized bases^2^

^§^ Pig No. 23 is still alive as of the preparation of this manuscript.

^1^ A frame-shifting indel introduces a stop codon 47 amino acids downstream in A403GfsX47 (sequence presented Fig 2)

^2^ Incompletely humanized R408W.

The initial Ossabaw piglet from Pregnancy A (Fig 1c, middle left panel) was slightly below average weight (Fig 1d, 464 g vs 650 g) and demonstrated marked hypopigmentation compared to wild type Ossabaw piglets (Fig 1c, left panel), characteristic of disrupted phenylalanine metabolism. Plasma from cord blood showed elevated phenylalanine at birth (247 μM compared to 70 μM for the wild type sow). The piglet was fed unmodified colostrum replacer for the first 24 hours, at which point, neurological dysfunction developed, manifesting as lethargy, poor feeding, and ataxia. A peripheral blood sample taken at 24 hours showed a rise in plasma phenylalanine to 937 μM. Treatment with 20 kcal/oz Phenex-1 (Abbott Laboratories, Abbott Park, IL), a modified amino acid/low-phenylalanine milk replacer used for human PKU patients, was initiated at 24 hours after birth. Within the next 6 hours the piglet showed increased activity levels and normalization of neurologic function. Treatment with Phenex-1 continued until 80 hours old, when the piglet developed scours (possibly related to dietary transitions) and ultimately failed to thrive. Post mortem plasma analysis showed phenylalanine levels had dropped to 150 μM, suggesting that treatment with Phenex-1 had been able to normalize blood phenylalanine.

Subsequent SCNT efforts transitioned to the Yorkshire background, a commonly cloned breed that is not predisposed to metabolic abnormalities that are observed in the Ossabaw background [18, 19]. This resulted in 9 live piglets from 4 pregnancies (4, 1, 2, and 2 piglets, respectively, Table 1). The four piglets from Pregnancy B (Fig 1c, middle right panel) were approximately 75% of historical wild type body weight (Fig 1d), but were otherwise phenotypically unremarkable at birth. Piglets 1795 and 1796 were robust at birth and independently fed well on colostrum replacer until 36 hours, at which point, severe neurological dysfunction (pedaling, ataxia, epilepsy) was observed and both piglets succumbed to lethal seizures around 40 hours of age. It was unknown if the seizures were related to the metabolic phenotype or possible dehydration from nutritional diarrhea. Piglet 1794, similar to 1769, started showing neurological symptoms (ataxia, lethargy, walking in circles) after consuming colostrum replacer for 24 hours. Treatment with Phenex-1 ameliorated neurological symptoms within four hours, further validating the fidelity of this model to the human disease phenotype, but nutritional diarrhea ultimately led to the death of this piglet around 100 hours of age. Piglet 1797 consumed colostrum replacer for the first 24 hours before treatment with Phenex-1 began. This piglet never fed well independently and ultimately died due to complications of failure to thrive. Post mortem plasma analysis showed moderately elevated phenylalanine in Piglet Nos. 1794–1797 of 349, 341, 274, and 477 μM, respectively.

Pregnancy C resulted in a single live piglet (No. 1798). This animal had the expected elevated cord blood phenylalanine at 345 μM. With the hypothesis that earlier treatment with Phenex-1 would prevent the onset of neurologic dysfunction and reduce the occurrence of nutritional diarrhea present in the previous litters, Piglet No. 1798 was fed a mixture of colostrum replacer and Phenex-1 at a ratio of 9:1 for the first six hours of life, 3:1 for hours six through 18, 1:1 for hours 18 through 24, and Phenex-1 alone from 24 hours on. No neurological dysfunction was observed, but the onset of mild diarrhea began around 24 hours of age and progressively worsened despite intraperitoneal fluid therapy and treatment with antibiotics. Ultimately, death occurred around 105 hours. Interestingly, post mortem plasma phenylalanine was within normal limits at 133 μM, suggesting 1) early treatment with Phenex-1 was able to normalize blood phenylalanine and 2) the lethality observed through this pregnancy was not likely related to the phenotype of hyperphenylalaninemia.

**Fig 2:**
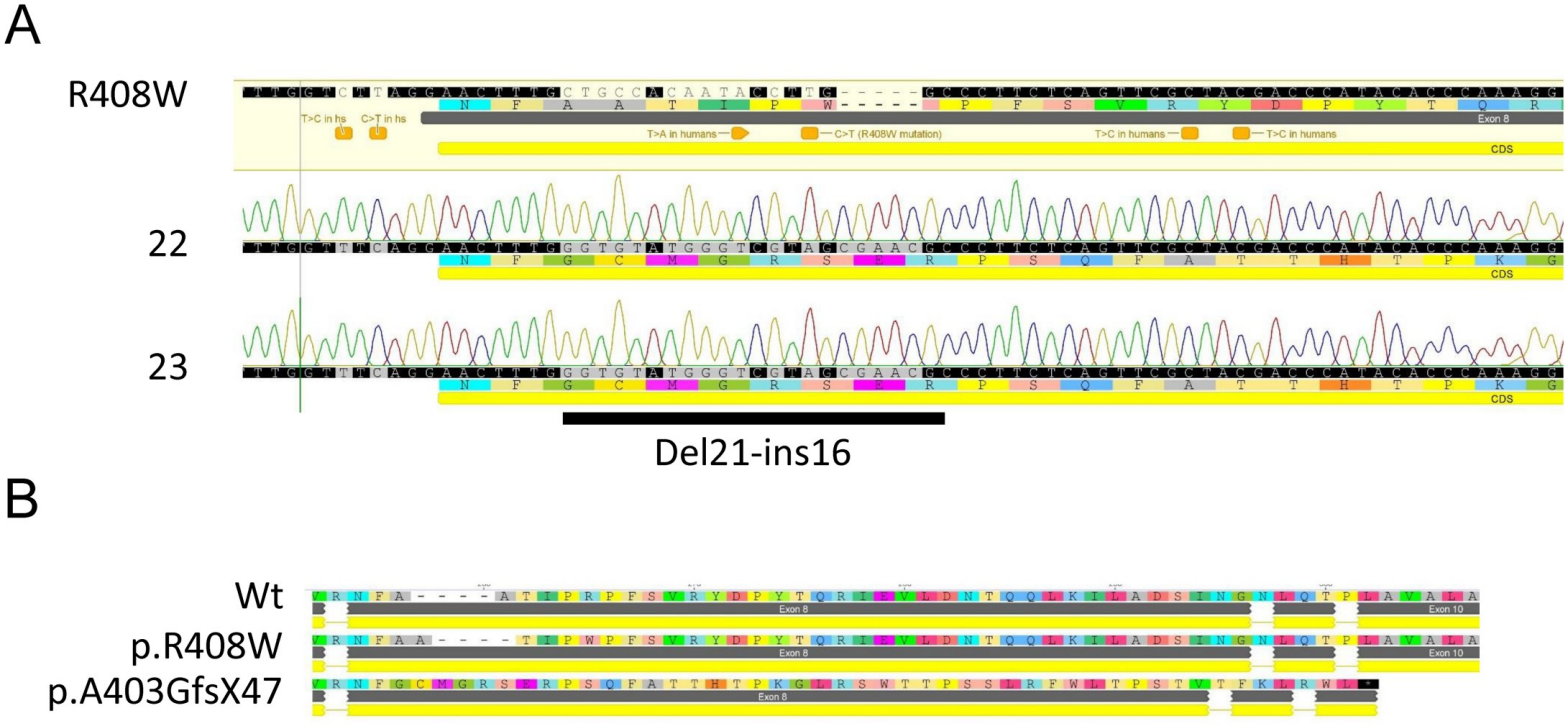
Genotype of compound heterozygous hR408W/pA403GfsX47 pigs. A) Sanger sequencing of TOPO clones from Piglet Nos. 22 (Charm) and 23 (Lucky). Only the non-R408W allele is shown, revealing an indel consisting of a 21 base pair deletion with a 16 base pair insertion. B) Translation of the indel cDNA and alignment the wild type and R408W shows the frame shift mutation begins at amino acid A403 resulting in a stop codon 47 amino acids downstream. This allele does not produce functional PAH, as predicted by this translation.

**Fig 3:**
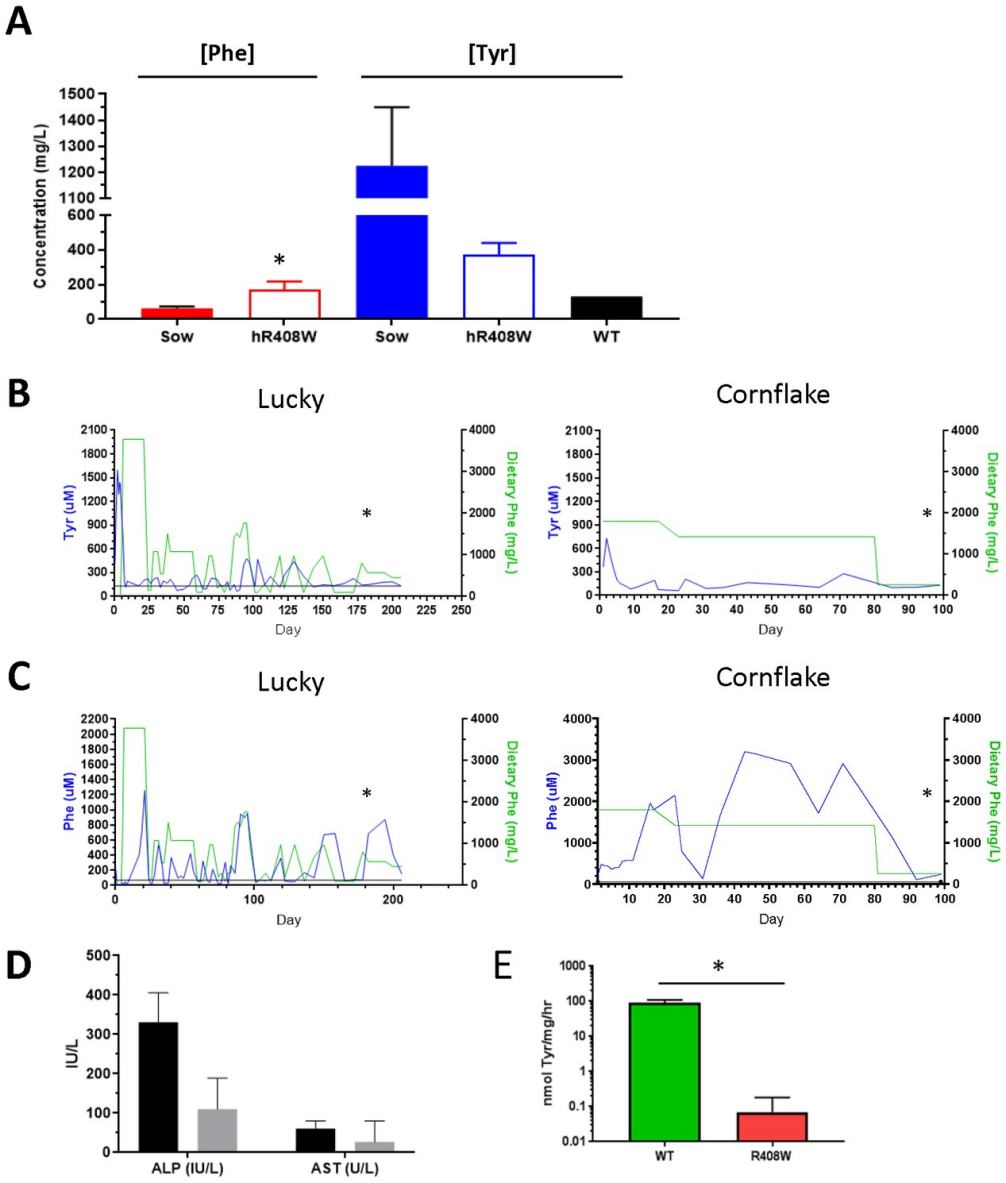
*PAH*-targeted phenotype characterization. A) Yorkshire founder PKU piglets maintained on NTBC were analyzed at birth for circulating phenylalanine and tyrosine levels, compared to the sow (assayed a C-section). Wild type tyrosine level is represented for comparison due to the elevated levels present in the sow, as maintained on NTBC. B) Two piglets (Lucky and Cornflake) were maintained continuously to characterize the R408W/x metabolic phenotype. Tyrosine levels (blue) were elevated at birth due to NTBC administration during gestation, and showed muted responsiveness dietary phenylalanine available during development (green). C) Serum phenylalanine levels (blue) were well correlated with dietary phenylalanine (green), indicative of disrupted phenylalanine metabolism, modeling human disease. Asterisk represents timing of clinical chemistry analysis in the next panel. D) Liver enzyme analysis of the long term piglet (Lucky) showed healthy ALP and AST levels while maintained on phenylalanine-restricted diet. E) PAH activity was assayed from liver homogenates of animals harvested within 1 week of birth demonstrating nearly complete ablation of activity despite expression, indicating loss of function from the hR408W mutation. * p<0.05 compared to WT sow (A) or age matched control (E).

For Pregnancies D and E, the sows were maintained on 2-(2-nitro-4-trifluoromethylbenzoyl)-1,3-cyclohexanedione (NTBC, the drug used to treat tyorisinemia type 1) at a daily dose of 25 mg to inhibit tyrosine metabolism, with the hypothesis that any gestational effect of *PAH*-targeting could cause hypotyrosinemia [20] from lack of PAH activity. Therefore, this pharmacological prevention of metabolism of residual tyrosine may help support proper early development. These four piglets, (two animals shown in Fig 1c, right panel) were phenotypically unremarkable at birth, with body weights from 1120 to 1630 grams (Fig 1d, hR408W NTBC), statistically higher than untreated hR408W counterparts, and had phenylalanine levels of 97 to 214 μM (Fig 3a). Surviving piglets in Pregnancies D and E were compound heterozygotes featuring one fully targeted hR408W allele, as well as a second *PAH*-null allele variety. In Pregnancy D this was a frame-shifted indel that introduced a stop codon 47 amino acids downstream (*PAH*^hR408W/A403GfsX47^, Fig 2). In Pregnancy E this was a partially humanized R408W, where the first two 5’ SNPs were not humanized (*PAH*^hR408W/R408W^). One piglet from each litter (22 and 900, subsequently named Charm and Cheerio) had a similar demise as previous litters and died on day 6 of life, but another piglet from each litter (23 and 899, subsequently named Lucky and Cornflake) survived the neonatal period and were maintained for chronic phenotypic characterization. These piglets all showed elevated tyrosine at birth due to gestational NTBC administration (Fig 3a), which declined to normal levels after birth since the piglets were not maintained on NTBC (Fig 3b) post-delivery.

Diet for Lucky and Charm was modified dynamically during the neonatal period, aimed at addressing transient diarrhea while also providing sufficient nutrition in the context of metabolic disease (Fig 3b and 3c, green line, left panels). This regiment consisted of colostrum (200 mls each) for the first 7 hours, Phenex-1 starting at hour 8 until day 3, 50% Phenex-1/50% Birthright milk replacer on days 3–6, and 100% Birthright milk replacer from day 6 until week 3 of life. Lucky showed increasing phenylalanine levels until dietary phenylalanine intake was restricted at 3 weeks of age, and the diet was continually modified thereafter to target phenylalanine levels of 100–300 μM. Diet for Cornflake and Cheerio was not modified, and both piglets received normal colostrum replacer for up to 48 hours, pig milk replacer until weaned, and then normal feed (Fig 3b and 3c, green line, right panels). Cornflake showed chronically elevated phenylalanine levels, with fluctuations likely related to growth needs and proximity of blood collections with food consumption. On Day 80 we reduced dietary phenylalanine in Cornflake due to severe neuromotor ataxia resulting in abrasions to the lower extremities. Good association was shown between dietary phenylalanine intake and circulating phenylalanine levels in both Lucky and Cornflake (Fig 3c) consistent with absent PAH activity and consequences of the human PKU phenotype despite the slight variations in the compound heterozygous genotype. Analysis of serum alkaline phosphatase and aspartate aminotransferase (AST) concentrations from Lucky and Cornflake indicated overall good liver health in the long-term piglets (Fig 3d). However, during treatment for the ataxia complications with wound care led to euthanasia for humane purposes.

Post mortem analyses of the piglets that did not survive past 7 days showed negligible PAH enzymatic activity compared to wild type, falling below the level of detection for all *PAH*-targeted piglets tested (Fig 3e). This radiometric assay can reliably detect phenylalanine conversion down 1% of the rate in wild type liver. This result is similar to that expected for the human phenotype, where the R408W mutation achieves complete ablation of PAH activity despite seemingly unaltered levels of expression via immunofluoroscopy (S1 Fig).

Amino acid analysis of lysates of brain cortex from *PAH*^hR408W/hR408W^ piglets and from wild type controls (Table 2) showed higher levels of phenylalanine in the brain of *PAH*^hR408W/hR408W^ piglets, similar to the pattern present in wildtype and *Pah*^enu2/enu2^ mice and demonstrating the expected correlation with blood phenylalanine concentrations. Additionally, *PAH*^hR408W/hR408W^ piglets that consumed colostrum replacer alone had higher levels of both blood and brain phenylalanine than piglets that were fed Phenex-1, further demonstrating the translatability of this model to the human disease. Measurement of monoamine neurotransmitters was attempted retrospectively on postmortem cerebral cortex samples from Piglet Nos. (1794, 1795, 1796, 1797, and 1798 (S1 Table). Although monoamine neurotransmitter deficiency is expected in hyperphenylalaninemic animal models [21], interpretation of the data from these initial animals is limited due to 1) post mortem collections of cerebral cortex at indeterminate intervals as the animals died or were euthanized, and 2) lack of contemporaneous wild type control samples collected under similar conditions. Future evaluations will include prospective and specific collection of substantia nigra and/or basal ganglia where the concentrations of monoamine neurotransmitters would be expected to be greater than in cerebral cortex. In general, the mean concentrations of dopamine, serotonin, and 5-HIAA were greater in piglets treated with low Phe diet than in animals that died while consuming only colostrum replacer, suggesting that lowering blood phenylalanine was associated with improved brain monoamine neurotransmitter status; however, the variability described above preclude definitive interpretation of this endpoint.

**Table 2:** Brain amino acid profiles in PAH^R408W/R408W^ piglets.

		WT	Normal Diet (n = 2)			Treated (Phenex 1, n = 3)				NTBC (n = 2)			C57Bl Mouse	*Pah*^*enu2/enu2*^ Mouse
		Ref	1795	1796	Mean ± SD	1794	1797	1798	Mean ± SD	21	23	Mean ± SD	Mean ± SD	Mean ± SD
	Age (hrs)	N/A	36	36	N/A	100	60	80	N/A	168	0		N/A	N/A
Amino acid (nmol/g wet weight)	Phe	221	767	539	653 ± 114	484	969	179	544 ± 325	550	350	450 ± 100	121 ± 66	771 ± 80
	Tyr	271	207	193	200 ± 7	385	281	210	292 ± 72	1989	668	1329 ± 660	73 ± 52	49 ± 40
	Trp	25	30	17	24 ± 6	95	51	42	63 ± 23	36	43	40 ± 3	21 ± 7.9	16 ± 7
	Ser	1355	872	694	783 ± 89	1683	1461	1131	1425 ± 227	2421	1915	2168 ± 253	1049 ± 89	1297 ± 73
	Ala	2188	509	660	584 ± 76	3002	1038	1451	1830 ± 846	6067	2784	4426 ± 1642	883 ± 160	829 ±127
	Pro	360	282	190	236 ± 46	1127	523	413	688 ± 314	1105	404	755 ± 351	216 ± 17	233 ± 12

Phe—phenylalanine; Tyr—tyrosine; Trp—tryptophan; Ser—serine; Ala—alanine; Pro—proline

**Fig 4:**
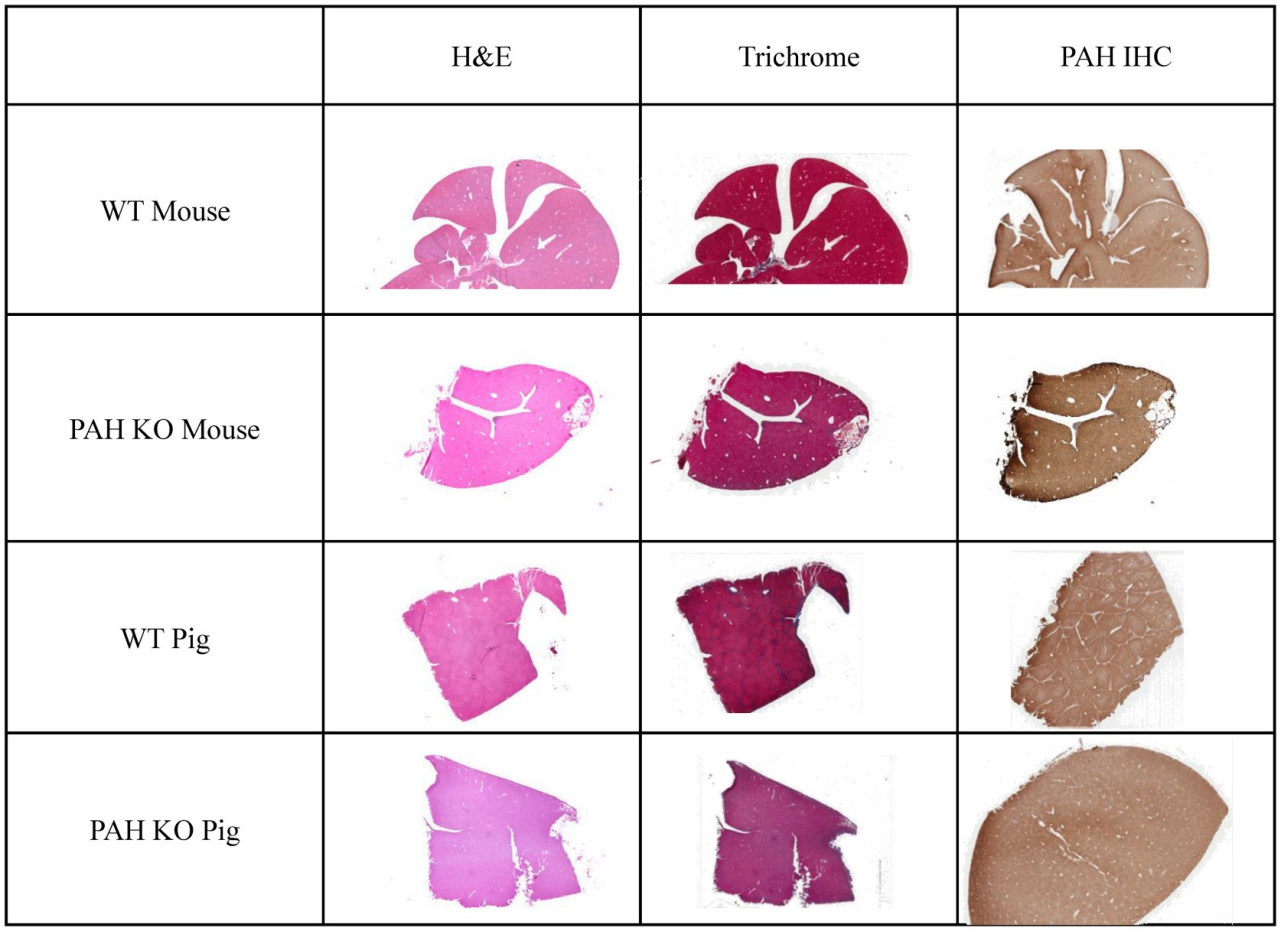
Histology of WT and *Pah* KO livers in mouse and pig. Standard H&E and Masson’s trichrome sections show no appreciable difference between WT and *Pah*^enu2/enu2^ mice or WT and *PAH*^R408W/R408W^ pigs. Similarly, immunohistochemistry showed similar levels and ubiquitous distribution of expression of the wild type and mutant protein in both models/species.

Histologically, there were no variations in liver morphology (H&E), fibrosis (Masson’s trichrome), or PAH expression patterns (IHC) in *Pah*-targeted or wild type livers of mice or pigs (Fig 4). Again, this is consistent with the human phenotype where PKU is not associated with pathology in the liver, and *PAH* expression is largely unaltered between WT and PKU alleles.

**Fig 5:**
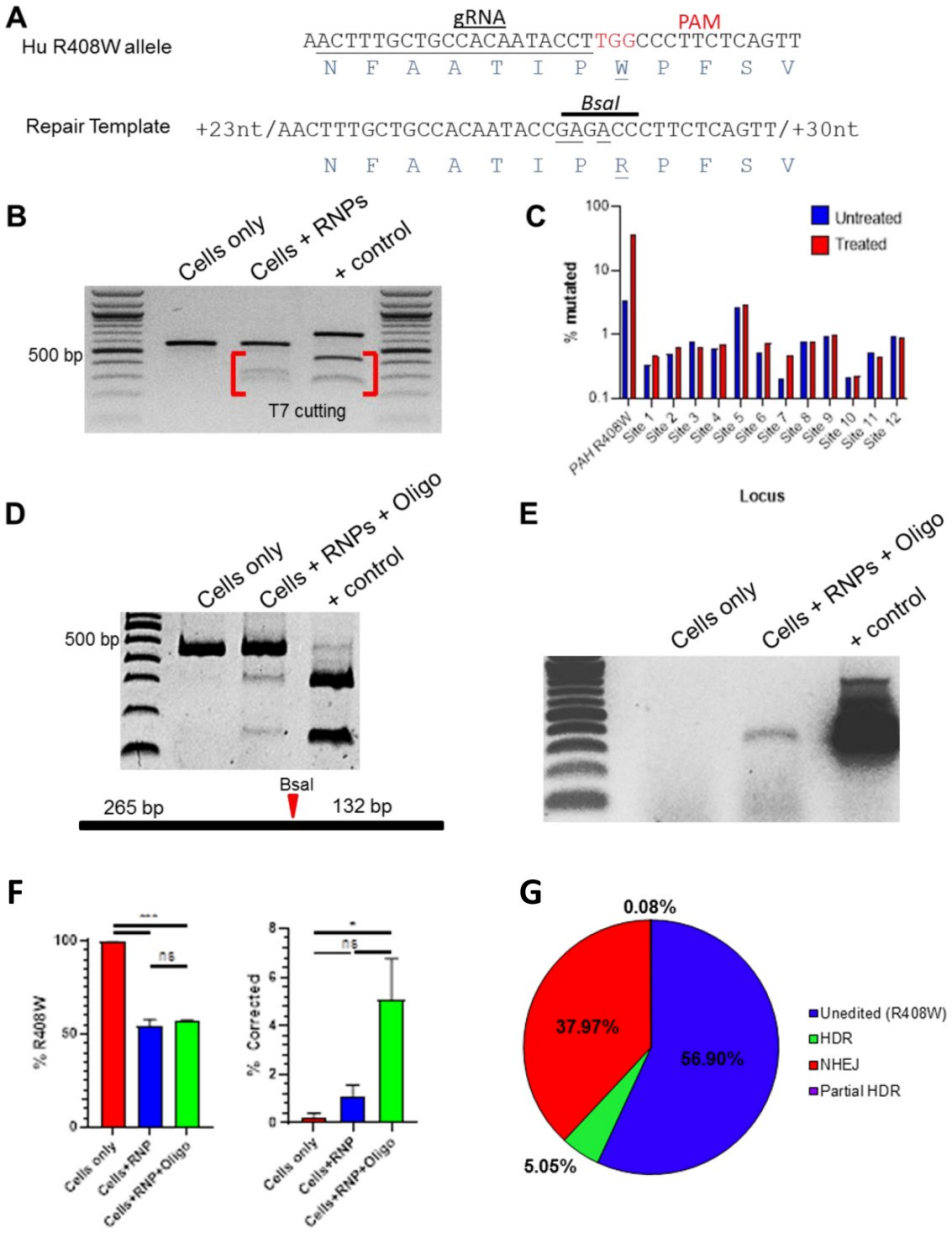
*In vitro* targeting of humanized PAH^R408W^ and correction by HDR. A) Sequence alignment of humanized pig *PAH*^hR408W^ (top) showing the guide RNA for the RNPs (grey arrow) as well as a homology template modified to revert the mutation and disrupt the PAM to prevent re-cutting (bottom). The location of the R408W mutation (red letters) and engineered BsaI site are also indicated. B) T7 endonuclease assay of *PAH* PCR products derived from hR408W fibroblasts, fibroblasts treated with RNPs containing the PAH gRNA, and a positive control for T7 cutting. The presence of the lower/lighter bands indicate mismatched double-stranded DNA indicative of NHEJ resulting from the RNPs. C) Quantitation of amplicon sequences derived from unedited (blue) and RNP treated (red) PCR of the *PAH*^hR408W^ locus as well as the top 12 predicted off-target cutting sites for the gRNA employed. Only the *PAH* locus showed indels above background detection levels when compared to untreated samples. D) RFLP with BsaI was negative for untreated hR408W fibroblasts, while the diagnostic bands were present when cells were co-transfected with the RNP and the single stranded oligo template for HDR. Positive control was based on a synthesized dsDNA encoding the intended HDR product. Relative position of the BsaI cut site is diagramed below the gel for reference. E) HDR-specific PCR showed the presence of the anticipated product in co-transfected cells, but not untreated hR408W fibroblasts. The positive control from D, synthetic DNA created to produce the target HDR sequence for analysis, was also evaluated. F) The predominant sequence in untreated cells was R408W, which is reduced in both RNP and RNP/HT co-transfected cells. G) Pie chart indicating the relative presence of unedited (R408W), NHEJ, HDR (W408R and BsaI site), and incomplete HDR (not all corrections present) species in PAH locus amplicon sequencing from panel F. Gel images presented in B, D, and E are from a single gel each that was abbreviated for presentation as indicated by black vertical lines.

The utility of this PKU model is enhanced by the ability to preclinically develop sequence-specific gene editing tools capable of being directly translated to human patients, including those with a classic R408W allele. To that end, a homology template (HT) to correct hR408W (Fig 5a) and associated Cas9 guides were designed to repair the mutant allele as proof of concept toward developing a potential therapeutic gene editing approach. Cas9 ribonucleoprotein complexes (RNPs) containing the R408W-targeting guide RNA were transfected into hR408W fibroblasts and assayed by T7 endonuclease for the introduction of indels. These RNPs induced indel formation at the *PAH* locus (Fig 5b and 5c), while analysis of the top 12 predicted off-target gRNA binding sites showed no substantial evidence of cutting by T7 (not shown) or amplicon sequencing relative to variation present in untreated controls (Fig 5c). The top 10 disruptions at *PAH* relative to the R408W mutation mostly included deletions of 1–14 bp (S2 Table). The unedited sequence was only the second most prevalent detected, indicating very efficient cutting and indel formation.

With this verification of the specificity of indel formation, RNPs and a single-stranded homology template (ssODN) were co-transfected into the same fibroblasts. The HT was further engineered to delete the PAM to prevent re-cutting and introduce a silent RFLP that would allow for efficient identification of HDR events (Fig 5a). HDR was confirmed at the population level by the presence of the RFLP in experimental samples (Fig 5d). Additionally, PCR designed using a 3’ primer specific for the HDR event (and a 5’ primer outside of the ssODN) amplified the target product in co-transfected cells, further indicating HDR had occurred (Fig 5e). While R408W was the primary sequence present in untreated fibroblasts, RNPs caused 50% reduction of the presence of the R408W sequence in amplicons sequenced from cells treated with or without ssODN (Fig 5f). Deep sequencing of the resulting amplicon showed disruption at 43% of the products present, with 5% being HDR and 38% NHEJ (Fig 5g). Together, these data indicate the efficacy of these RNPs to initiate HDR to correct R408W in humanized sequences in these fibroblasts.

The *PAH*-targeted piglets are fragile in the neonatal period. The fragility is difficult to completely ascribe to the PKU phenotype and maybe related to first generation clones seen in other pig models of human diseases [22, 23]. Therefore, since hyperphenylalaninemia is not acutely toxic in human patients, the emphasis in rearing was shifted from attenuating circulating phenylalanine levels to simply maintaining neonatal piglet health. Like patients, *PAH*^hR408W/hR408W^ pigs express mutant PAH similar to wild type protein levels in healthy pigs, and the protein product cannot metabolize phenylalanine. Expression of the mutant protein is useful to model unanticipated effects of this expression in experimental disease and therapy studies, but it also reduces the potential for immunogenicity against expression of a *PAH* transgene or the protein product of an edited *PAH* locus. In the case of R408W, function is eliminated by the substitution of a single amino acid in the transcript, which represents less than 0.3% variation relative to the wild type protein. This theoretical attenuation of immunogenicity would be anticipated to benefit enzyme replacement therapy as well as gene therapy approaches.

Although the utility of this animal to model PKU is valuable for basic research, a broader application of this animal model is the ability to show proof-of-concept for human gene editing platforms, regardless of the target indication. Human therapeutic gene editing in this pig is limited to the context of PKU, but the animal can serve as a model for testing multiple gene therapy platforms at this humanized locus. Indeed, this model can be used to assess safety and efficacy of delivery platforms, such as viral or nanoparticle systems, or the effectiveness of different editing platforms, either *in vitro* or *ex vivo*. This generalized use of the model will be useful for translating a variety of gene therapy investigational drugs into the clinic.

To our knowledge, this is the first large animal model to both mimic the disease phenotype, but also model the precise local genotype of a common disease allele. As with most rare genetic diseases, there is no available clinical cure, and current treatments only address the symptoms or themselves reduce the quality of life. Gene therapies, particularly somatic cell gene editing therapies, for many of these diseases represent a viable path to a cure. The ability to engineer allele-specific, and even patient-specific, mutations in large animal models will accelerate the development and improve the chances of success in clinical trials.

## Methods

### Animals

All procedures involving live animals were conducted in compliance with regulations outlined by the Institutional Animal Care and Use Committee of Cooperative Resources International (CRI) International Center for Biotechnology (IBC) and this study was approved by the Institutional Animal Care and Use Committee of Mayo Clinic. Where indicated for the study, euthanasia was performed by barbiturate overdose, an AVMA accepted method. PAH^R408W/R408W^ piglets were produced in both the Ossabaw and large white/landrace strains. Piglets were derived via cesarean section on day 118 of gestation and were hand-reared on a combination of commercially available bovine colostrum/milk replacers (Bovine IgG Calf’s Choice Total^®^ Gold, SCCL, SK, Canada; CL Sow Replacer, Cuprem^®^, Kenesaw, NE, USA; Birthright^™^, Ralco Animal Nutrition, Marshall, MN, USA) and a phenylalanine-free human infant formula (Phenex^®^-1, Abbot Nutrition, IL, USA).

### TALEN design and production

Candidate TALEN target DNA sequences and repeat variable diresidue (RVD) sequences were identified using the online tool “TAL EFFECTOR NUCLEOTIDE TARGETER 2.0”. Plasmids for *in vitro* TALEN mRNA transcription were then constructed by following the Golden Gate Assembly protocol using RCIscript-GOLDYTALEN (Addgene ID 38143) as final destination vector [24]. Assembled RCIscript vectors prepared using the QIAPREP SPIN MINIPREP kit (Qiagen) were linearized by SacI (NEB) to be used as templates for in vitro TALEN mRNA transcription using the mMESSAGE mMACHINE^®^ T3 Kit (Ambion) as indicated previously [25]. Resulting mRNA was DNase treated prior to purification using the RNeasy Kit (Qiagen).

### Tissue culture and transfection

Outbred Ossabaw and large white pig fibroblasts were briefly maintained at 38.5°C at 5% CO2 in DMEM supplemented with 10% fetal bovine serum, 100 I.U./mL penicillin and streptomycin, 2mM L-Glutamine and 10mM Hepes. Once fibroblasts reached 90% confluency, they were spilt 1:2 and harvested the next day. The Neon Transfection system (Life Technologies) was used to deliver the TALEN mRNA (500 ng each; *ssPAH* 8.1 L, *ssPAH* 8.1 R) and ssODN (0.2 nmoles; *ssPAH* R-W 90 (5’-TCTCAGATCTCTGGTTTTGGTCTTAGGA ACTTTGCTGCCACAATACCTTGGCCCTTCTCAGTTCGCTACGACCCATACACCCAAAGGATT-3’)). Approximately 600,000 cells were resuspended in “R” Buffer with mRNA TALENs and HDR oligo, and electroportated using the 100 μL tips and the following parameters: input voltage: 1800V; pulse width: 20 ms; pulse number: 1. Transfected cells were dispersed into one well of a 6-well plate with 2 mL DMEM media and cultured for 3 days at 30°C prior to population efficiency testing.

### Sample preparation

Transfected cell populations were collected. 50% of the cells were re-seeded onto one well of a 6-well plate with 2mL fresh DMEM growth media, 40% were resuspended in 80 μL cryopreservation media (90% FBS, 10% DMSO), and 10% were resuspended in 20 μL of 1X PCR compatible lysis buffer (10 mM Tris-Cl pH 8.0, 2 mM EDTA, 0.45% Tryton X-100(vol/vol), 0.45% Tween-20(vol/vol)) freshly supplemented with 200 μg/ml Proteinase K. The lysates were incubated in a thermal cycler using the following program: 55°C for 60 minutes, 95°C for 15 minutes.

### TALEN efficiency

PCR amplification was conducted using AccuStart^™^ Taq DNA Polymerase HiFi (Quanta Biosciences) with 1 μL of the cell lysate as template. The following primers and program were used: *ssPAH* E8 F1 (5’-CTTCACCTCTCAGCCTGGTC-3’) and *ssPAH* E8 R1 (5’-TGCCACGTTTCGTTCTCTCA-3’); 1 cycle (95°C, 2 minutes), 35 cycles of (95°C, 20 s; 62°C, 20 s; 68°C, 45 s), 1 cycle (68°C, 5 minutes). Frequency of mutations in the population was analyzed with the SURVEYOR MUTATION DETECTION Kit (Transgenomic) according to the manufacturer’s recommendations, using 10 μL of the PCR product. The products were resolved on a 10% TBE polyacrylamide gels and visualized by ethidium bromide staining. Densitometry measurements of the bands were performed using ImageJ; and mutation rate of SURVEYOR reactions were calculated as described previously [26].

### Single-cell derived clonal isolation and Chromatin Transfer

Four days post transfection, cells were seeded onto 10 cm plates at a density of 100 cells/plate and cultured until individual colonies reached approximately 5mm in diameter. Growth media was aspirated and the plates were washed with 4 mL PBS. 8 mL of a 1:4 (vol/vol) mixture of TrypLE and DMEM was added and colonies were aspirated in a volume of 150 μL, transferred into wells of a 48-well plate containing 150 μL DMEM growth media, mixed via manual pipetting and 150 μL was seeded into a replica 96-well plate and cultured at 38.5°C. The 96-well plates were incubated for 2 days prior to lysis (described above). PCR amplification using AccuStart^™^ II PCR SuperMix (Quanta Biosciences) was performed. Amplicons were purified using the QIAquick 96 PCR Purification Kit (QIAGEN) following manufacturer’s instructions and submitted for Sanger sequencing (ACGT, Inc.). Clones containing the desired genotype were cryopreserved in 70 μL cryopreservation media and submitted to International Center for Biotechnology, LLC. (formerly Cooperative Resources International) for Chromatin Transfer as described previously [27].

### Biochemical analysis

Blood was collected by venipuncture and processed to serum by centrifugintion at 1700 x g for 10 min at 4°C and analyzed for liver enzymes using a the VetScan VS2 benchtop analyzer (Mammalian Liver Profile, Abaxis, Union City, CA) according to the manufacturer’s instructions. Circulating Tyrosine and phenylalanine values were determined using tandem mass spectrometry and chromatography via Mayo Clinic’s internal biochemical PKU test.

### Western blot analysis

Western blotting was performed using an SDS-PAGE electrophoresis system. Homogenized liver samples were quantitated via Bradford assay, and 30-ug aliquots were resuspended in a reducing sample buffer, boiled and run on an 8% acrylamide reducing gel. Gels were blotted to PVDF membrane, and probed with PAH R400 polyclonal antibody (Bioworld Technology, #BS3704) at a dilution of 1:500. GAPDH (ThermoFisher #MA5-15738) was used as a loading control at a dilution of 1:50,000. Two secondary antibodies were used: an HRP-conjugated goat anti-rabbit antibody (Life Technologies #G21234) to bind to the PAH primary, and a goat anti-mouse antibody (Santa Cruz #sc-2055) for the GAPDH. Results were visualized on autoradiograph film using enhanced chemiluminenscence (SuperSignal West Pico Chemiluminescent).

### PAH enzyme activity assay

PAH activity was measured in duplicate on liver homogenates using a radiochemical technique [28] modified as previously described [29]. Briefly, total protein was measured using a bicinchoninic acid procedure (Microprotein Assay; Pierce, Rockford, IL, USA). Liver homogenates isolated from wild-type C57BL/6 mice were used as positive controls. PAH activity from piglet liver homogenates is expressed as a percentage of the wild-type PAH activity measured in mouse liver homogenates.

### Brain monoamine neurotransmitter analysis

Cerebral cortex biopsies were obtained postmortem, frozen and stored at -80°C until analysis. Biopsy specimens were thawed on ice and mechanically homogenized in ice-cold homogenizing buffer (50 mM Tris-HCl, pH 7.5, 0.1 M KCl, 1 mM EDTA, 1 mM dithiothreitol, 0.2 mM phenylmethylsulfonyl fluoride, 1 μM leupeptin, and 1 μM pepstatin), 4 μl/mg tissue and further processed according to a previously published method [30]. Monoamine neurotransmitter concentrations (dopamine, homovanilic acid (HVA), serotonin, and 5-hydroxyindoleacetic acid (5-HIAA)) were measured in brain tissue by HPLC and electrochemical detection [31]. Measured brain homogenate monoamine neurotransmitter concentrations were corrected for the protein content of the homogenate and expressed as nmol/g protein.

### Immunohistological analysis

Liver samples were fixed in 10% neutral buffered formalin (Protocol, Fisher-Scientific, Pittsburgh, PA) and processed for paraffin embedding and sectioning. For hematoxylin and eosin staining, slides were prepared with standard protocols. PAH immunohistochemistry using a polyclonal rabbit anti-PAH primary antibody (ab178430, Abcam, Cambridge, MA) was performed with a Bond III automatic stainer (Leica, Buffalo Grove, IL) with a 20-min antigen retrieval step using Bond Epitope Retrieval Solution 2 (Leica, Buffalo Grove, IL), and stained with diaminobenzidine (Leica, Buffalo Grove, IL).

### Fibroblast isolation, cell culture, and transfection

Primary PKU pig fibroblast cells were derived from neonatal Pah^-/-^ pig tissue and were kept in Dulbecco’s modified Eagle’s medium (DMEM; Thermo Fisher Scientific, Wal-tham, MA) containing 10% heat-inactivated fetal bovine serum (Corning, Herndon, VA) and 1% penicillin/streptomycin (Corning, Inc., Herndon, VA). All cells were kept at 37°C and 5% CO2. PAH fibroblasts were transfected with PAH sgRNA complexed with Alt-R^®^ S.p. Cas9 protein, guide RNA (5’-ACTTTGCTGCCACAATACCT) with or without HDR oligo (5’-G*A*TCTCTGGTTTTGGTCTTAGGAACTTTGCTGCCACAATACCGAGACCCTTCTCAGTTCGCTACGACCCATACACCCA*A*A*, where * represents phosphorothioate bond end-modifications) (IDT, Inc., Coralville, IA) [32]. Transfected cells were cultured for 2 days at 37°C prior to lysis. Total cells from in vitro assays were collected and DNA was purified using a Puregene Core Kit A (QIAGEN, Hilden, Germany) or DNeasy Blood and Tissue Kit (QIAGEN). Lysates were amplified by PCR and the subsequent analysis.

### PCR and T7 endonuclease analysis

For PCR amplification of Cas9-disrupted sequence, the following primers were used: 5’-ccttactaccttctgggcttt, 5’-cagggaaattctggcctttatg. PCR amplification was conducted using Gotaq polymerase (Promega) as follows: 95°C, 2 minutes; 30 cycles of 95°C, 30 seconds, 60°C, 30 seconds, 72°C, 40 seconds; 72°C, 10 minutes. Products were ~552 bp in length. The sequences were processed using a T7 endonuclease Alt-R Genome Editing Detection Kit (IDT, Inc., Coralville, IA).

### Off-target sites and correction analyses

Off-target sites were selected using Cas-OFFinder [33] using parameters of up to three mismatches and zero bulges. Fourteen sites were identified. One site was eliminated due to mismatch between the target cell line and the published library. One site was not successfully amplified. The remaining twelve sites (Table 3) were amplified with the following primers using Phusion^®^ HiFi DNA Polymerase (New England BioLabs, Ipswich, MA):

**Table 3:** Off target analysis sites evaluated for indel formation after CRISPR editing.

No.	Chromosome	Position	Strand	Forward Primer (5’->3’)	Reverse Primer (5’->3’)
**1**	AEMK02000361.1	1445594	-	CCTGAGAGCTGACACTGAGA	TTCACCCACTGATCAAGGCT
**2**	CM000813.5	3450748	+	CAAGTTCTCCTCCTCCCCTG	CTACAGATGCGGGAAAGAGC
**3**	CM000830.5	4607287	-	GGATTATGGCCCCAAAGCTG	TTAATACCCACATGCCCCGT
**4**	CM000820.5	9674178	+	GCACAAAGTAAGATCGCGCT	GATTCGGCGTCTTTCACCTG
**5**	CM000827.5	35596040	-	CAGCTCTTTTCTCCACCCCT	AGGTAGGGCAGGAGTTTCAC
**6**	CM000817.5	41976170	-	AGATCCACAACTGTTCTCCCT	TGGCACTACGACTTCACACT
**7**	CM000819.5	42290815	+	AGCTAAGCGATTGTCCATTGT	TCCTGCCAATGGTACTGACT
**8**	CM000829.5	47630943	+	GGTTCTCCAGCTCTTTTGTGA	TCAGTTGCTTCGGGTCTTTT
**9**	CM000827.5	57100262	+	GCAGAGAATTTCCACACCCA	TCTCCTCCTGTAGTTGCCAC
**10**	CM000824.5	66596456	+	CGCATAGTCCAGGTGAAGGT	CCTCACTCCTCAGGGCTATG
**11**	CM000816.5	81453668	+	CCAAGACTGTCTCCCTGGAA	CAGGACCAACATCTGTCTGC
**12**	CM000820.5	97228973	+	CAGAGTTGGCTTGGCTTTGG	TCCGGATGTGAACTAAGCCA

PCR products were analyzed using GENEWIZ’ Amplicon-EZ service (GENEWIZ, South Plainfield, NJ). Reads with fewer than five counts were not analyzed. Only changes within ten base pairs of the predicted cut site were considered to be mutations driven by Cas9.

For selective amplification of corrected sequence, the following primers were used: 5’-gaactgagaagggtctc (for binding selectively to the corrected sequence) and 5’-ccttactaccttctgggcttt (for binding outside of the homology region). Only DNA that had successfully incorporated the ssODN would amplify by this method. PCR conditions were as follows: 95°C, 2 minutes; 30 cycles of 95°C, 30 seconds, 60°C, 30 seconds, 72°C, 30 seconds; 72°C, 10 minutes. Products were ~322 bp in length. PCR and T7 products were electrophoresed on a 2% Tris-acetate-ethylenediamine tetraacetic acid buffer agarose gel and visualized with ethidium bromide staining.

For unbiased amplification of the corrected sequence locus, the following primers were used with Phusion^®^ HiFi DNA Polymerase (NEB, Ipswich, MA): 5’-acgttttggagggagatggt and 5’-acggaagaatggcctcaaag. PCR parameters were as follows: 95°C, 3 minutes, 37 cycles of 98°C, 10 seconds, 60°C, 20 seconds, 72°C, 15 seconds; 72°C, 5 minutes. Products were ~397 bp in length and were electrophoresed on a 2% Tris-acetate-ethylenediamine tetraacetic acid buffer agarose gel and visualized with ethidium bromide staining. Ten base pairs on either side of the predicted Cas9 cut site were analyzed for complete, partial, or no homologous recombination.

### PCR and Restriction Fragment Length Polymorphism (RFLP)

To further verify the correction, PCR was performed with the following primers: 5’-acgttttggagggagatggt, 5’-acggaagaatggcctcaaag. PCR conditions were as follows: 95°C, 2 minutes; 30 cycles of 95°C, 30 seconds, 60°C, 30 seconds, 72°C, 30 seconds; 72°C, 10 minutes. Products were digested with BsaI. 165bp and 232bp products were visualized on 10% TBE Gel with SYBR^™^ Safe DNA Gel Stain (both from Invitrogen, Cartsbad, CA).

## Supporting information

S1 Fig PAH expression analyses. A) Immunohistochemistry of liver for PAH expression in *PAH*^hR408W/hR408W^ Piglet No. 1769 (left), counterstained with Hoechst to identify nuclei. Wild type pig PAH expression in liver is presented for comparison (right). B) Immunohistochemistry of liver for PAH expression in *PAH*^hR408W/hR408W^ Piglet No. 1795 (left), wildtrype pig (middle) and wild type mouse (right) showing similar intensity of expression/staining for R408W and wildtype PAH. C) Western blot analysis shows that a 54 kDa PAH monomer (mutant/inactive) was detected in all *PAH*^*R408W/R408W*^ piglets in amounts similar to that of wild type large white pig liver. Muscle homogenate is presented as a negative control for PAH.
(TIF)

S1 Table Measurement of monoamine neurotransmitters was performed retrospectively on postmortem cerebral cortex samples from select piglets (1794, 1795, 1796, 1797, and 1798). However, these samples were collected post-mortem from animals with that succumbed spontaneously under variable husbandry, diet, dispositions and death details. These data are provided for completeness, but were not considered in evaluation of the model.
(DOCX)

S2 Table Fibroblasts from Pig No. 21 were transfected with Cas9 RNPs with a guide RNA targeting the R408W allele. Amplicon sequencing was used to characterize the top 10 most frequent PCR products at the *PAH* locus showing frequency of indel formation. Bold letters in WT sequence indicate humanized SNPs. The R408W codon is underlined.
(DOCX)

S1 Raw images (PDF)
