# Supplementary figures and images for: Development of a porcine model of phenylketonuria with a humanized R408W mutation for gene editing
Source: PLoS One. 2021 Jan 25;16(1):e0245831. doi: 10.1371/journal.pone.0245831 (PMC7833140; doi:10.1371/journal.pone.0245831)

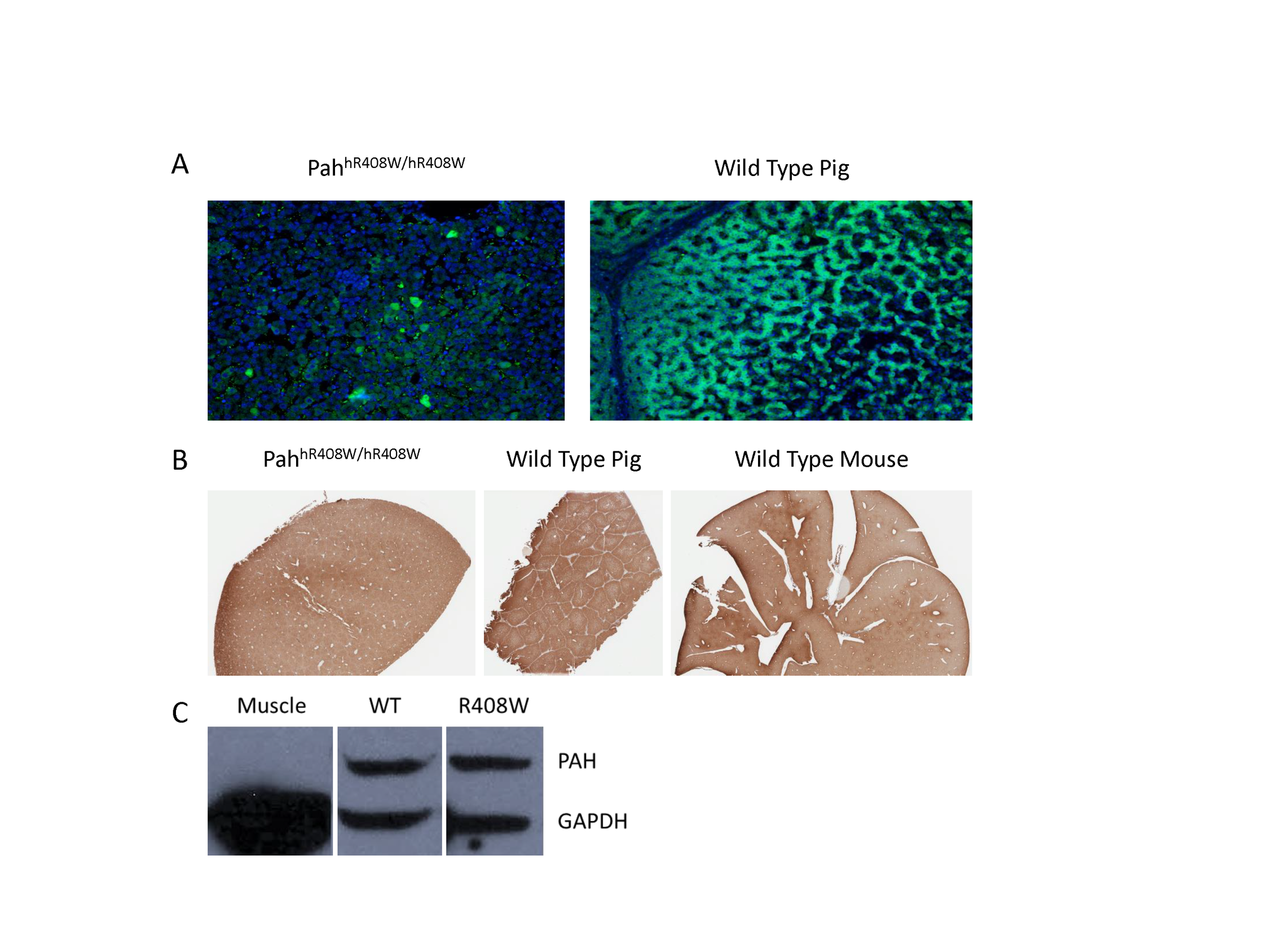

Supplement: S1 Fig — A) Immunohistochemistry of liver for PAH expression in PAHhR408W/hR408W Piglet No. 1769 (left), counterstained with Hoechst to identify nuclei. Wild type pig PAH expression in liver is presented for comparison (right). B) Immunohistochemistry of liver for PAH expression in PAHhR408W/hR408W Piglet No. 1795 (left), wildtrype pig (middle) and wild type mouse (right) showing similar intensity of expression/staining for R408W and wildtype PAH. C) Western blot analysis shows that a 54 kDa PAH monomer (mutant/inactive) was detected in all PAHR408W/R408W piglets in amounts similar to that of wild type large white pig liver. Muscle homogenate is presented as a negative control for PAH. (TIF) [file pone.0245831.s001.tif]

# Figure 4B source gel

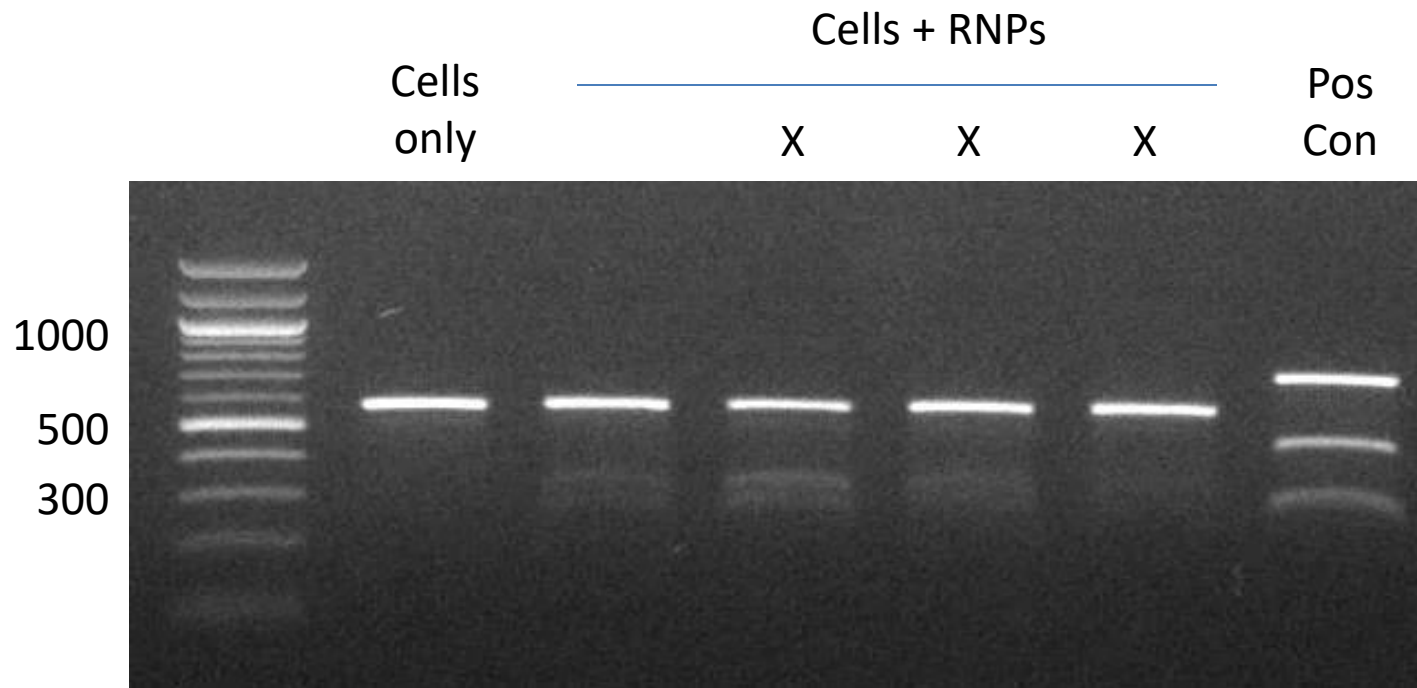

# Figure 4D source gel

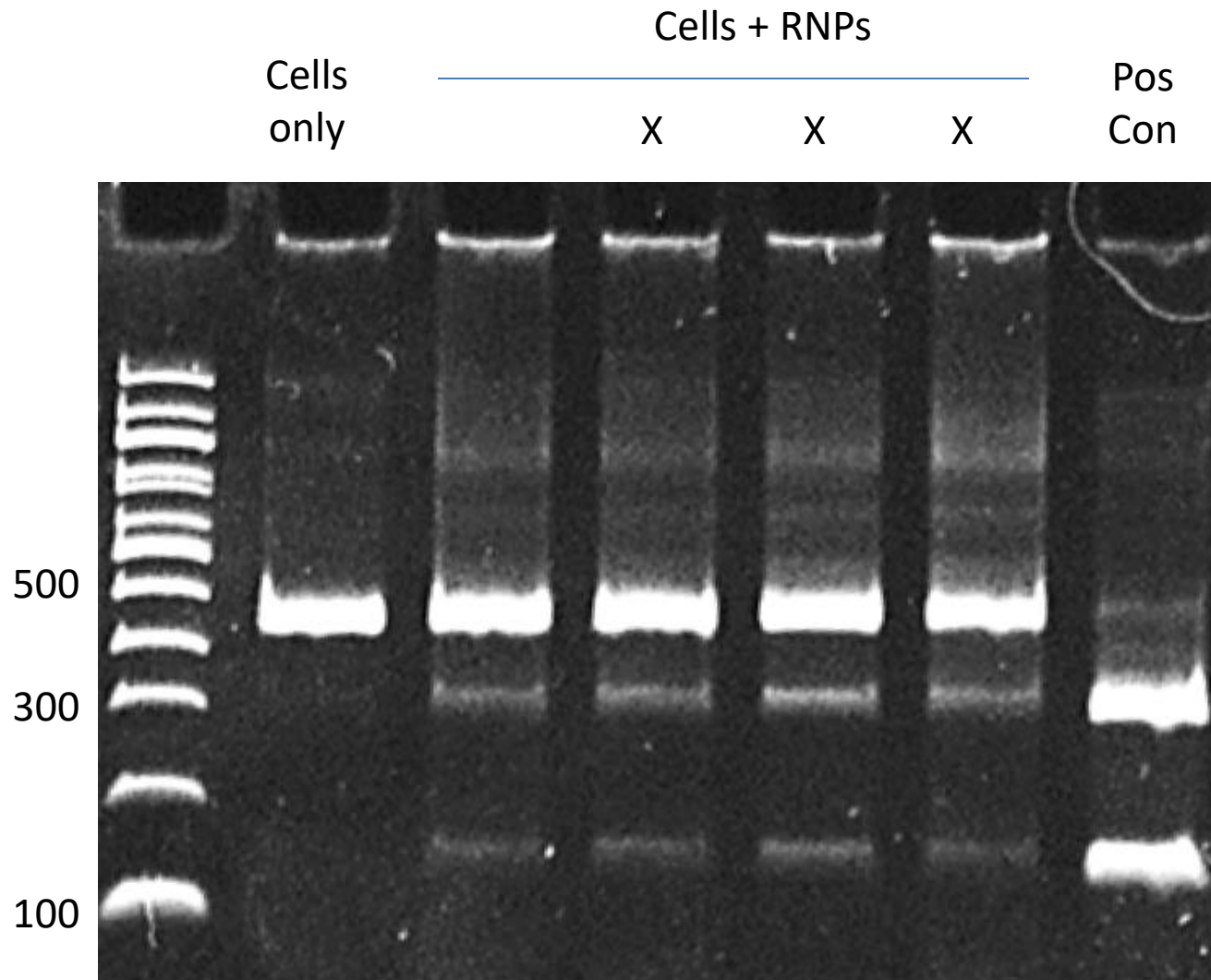

# Figure 4E source gel

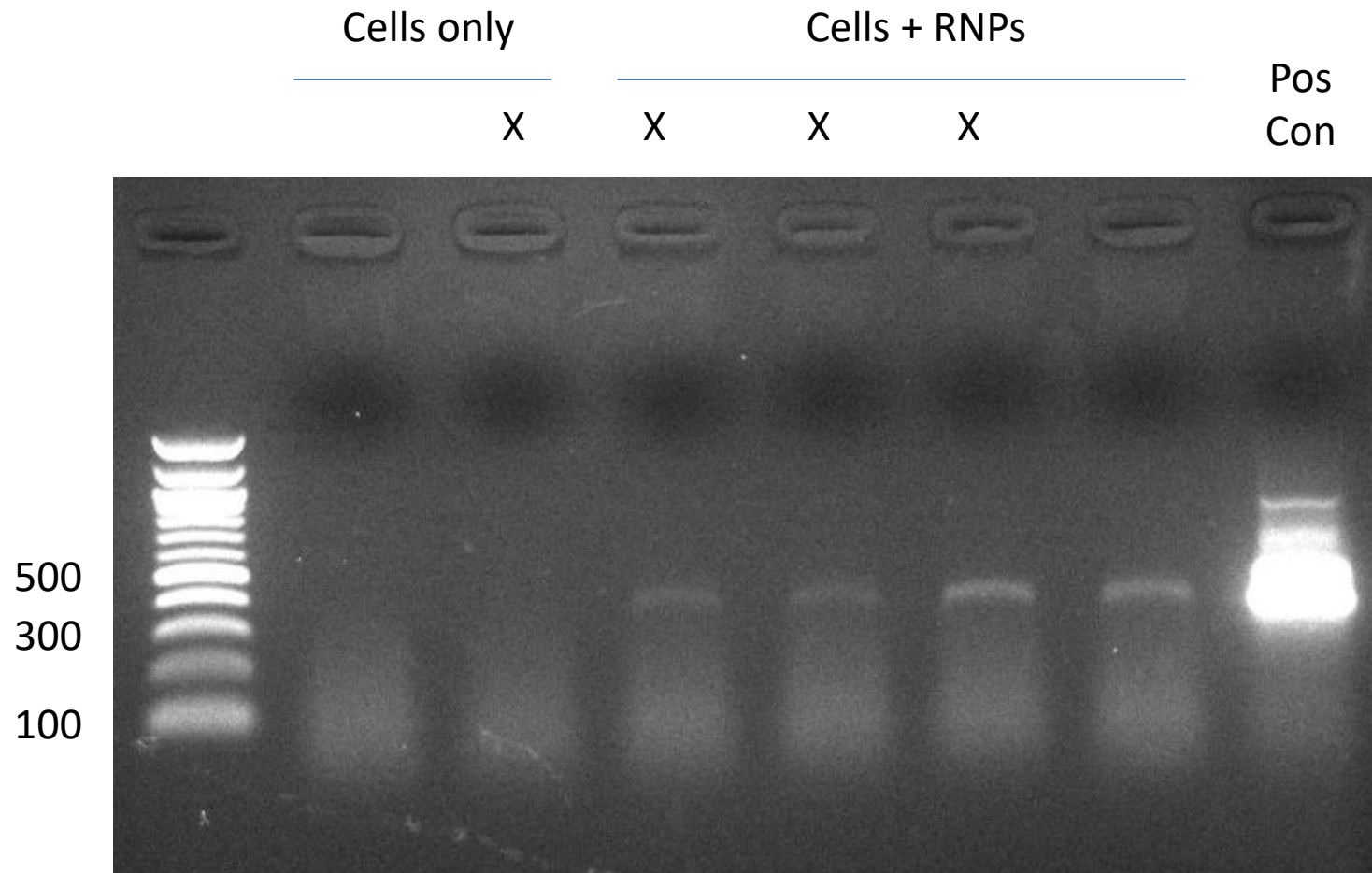

# Supplemental Figure 2 Source Blot

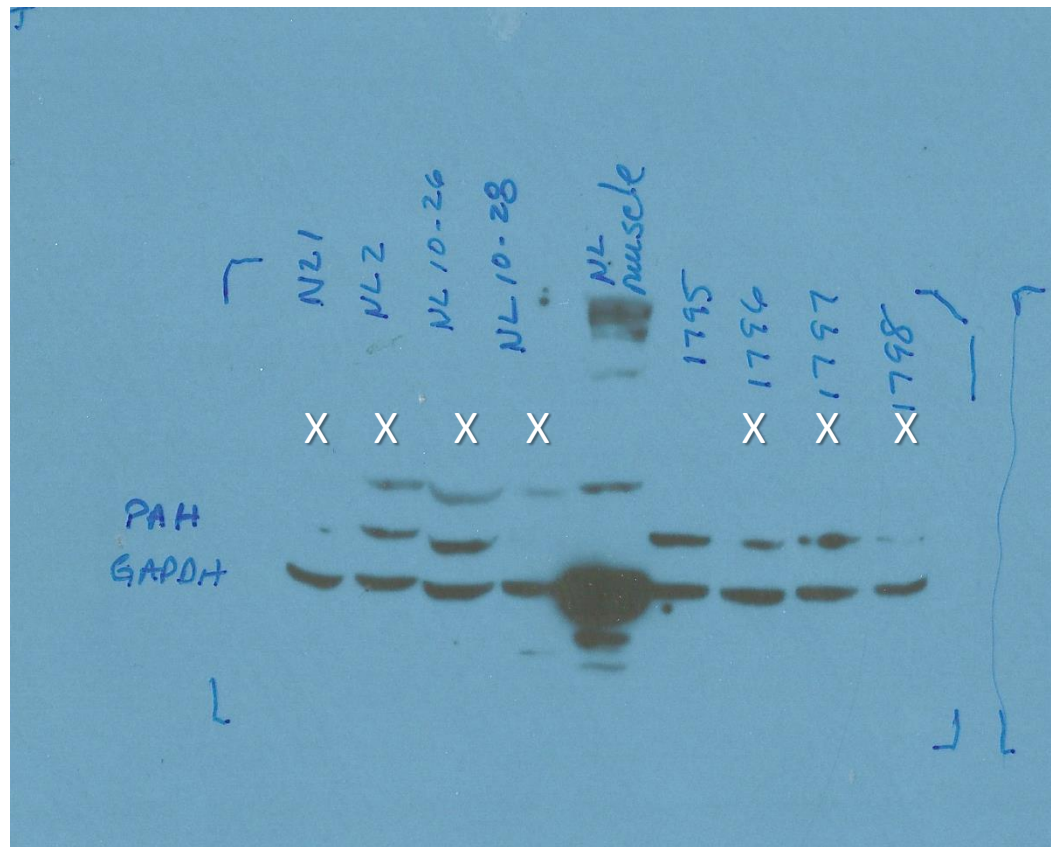

Supplement: S1 Raw images — (PDF) [file pone.0245831.s004.pdf]
