# Supplementary material for: Development of a porcine model of phenylketonuria with a humanized R408W mutation for gene editing
Source: PLoS One. 2021 Jan 25;16(1):e0245831. doi: 10.1371/journal.pone.0245831 (PMC7833140; doi:10.1371/journal.pone.0245831)
Supplement: S1 Table — However, these samples were collected post-mortem from animals with that succumbed spontaneously under variable husbandry, diet, dispositions and death details. These data are provided for completeness, but were not considered in evaluation of the model. (DOCX) [file pone.0245831.s002.docx]

**S1 Table. Brain neurotransmitters in select PAH-deficient piglets**

| **Neurotransmitter**  (nmol/g protein) | **Untreated** | | | | **Low Phe diet-treated** | | | | |
| --- | --- | --- | --- | --- | --- | --- | --- | --- | --- |
|  | 1795 | 1796 | Mean | SD | 1794 | 1797 | 1798 | Mean | SD |
| Dopamine | 15.47 | 0.14 | *7.80* | *10.84* | 9.94 | 37.57 | 258.75 | *102.09* | *136.37* |
| Homovanillic acid (HVA) | ND | ND |  |  | ND | 5.16 | 9.99 |  |  |
| Serotonin | 33.05 | 14.10 | *23.58* | *13.40* | 107.48 | 63.62 | 94.83 | *88.64* | *22.57* |
| 5-hydroxyindoleacetic acid (5-HIAA) | 64.05 | 53.56 | *58.81* | *7.42* | 114.39 | 53.92 | 80.98 | *83.10* | *30.29* |

ND – not detected
