# Supplementary material for: Development of a porcine model of phenylketonuria with a humanized R408W mutation for gene editing
Source: PLoS One. 2021 Jan 25;16(1):e0245831. doi: 10.1371/journal.pone.0245831 (PMC7833140; doi:10.1371/journal.pone.0245831)
Supplement: S2 Table — Amplicon sequencing was used to characterize the top 10 most frequent PCR products at the PAH locus showing frequency of indel formation. Bold letters in WT sequence indicate humanized SNPs. The R408W codon is underlined. (DOCX) [file pone.0245831.s003.docx]

| **Rank** | **Sequence** | **Count** | **%** |
| --- | --- | --- | --- |
| **R408W** | ggt**C**t**T**aggaactttgctgccacaat**A**cct**T**ggcccttctcagttcg**C**ta |  |  |
| **1** | GGTCTTAGGAACTTTGCTGCCACAAT**-**CCTTGGCCCTTCTCAGTTCGCTA | 19052 | 21.45 |
| **2** | GGTCTTAGGAACTTTGCTGCCACAATACCTTGGCCCTTCTCAGTTCGCTA | 10506 | 11.83 |
| **3** | GGTCTTAGGAACTTTGCTGCCACA**--**ACCTTGGCCCTTCTCAGTTCGCTA | 6919 | 7.79 |
| **4** | GGTCTTAGGAACTTTGCTGCCACAATA**-**CTTGGCCCTTCTCAGTTCGCTA | 3468 | 3.90 |
| **5** | GGTCTTAGGAACTTTGCTG**--------**CCTTGGCCCTTCTCAGTTCGCTA | 2648 | 2.98 |
| **6** | GGTCTTAGGAACTTTGCT**--------------**GCCCTTCTCAGTTCGCTA | 2575 | 2.90 |
| **7** | GGTCTTAGGAACTTTGCTGCCACAATA**A**CCTTGGCCCTTCTCAGTTCGCTA | 2561 | 2.88 |
| **8** | GGTCTTAGGAACTTTGCTGCCAC**---**ACCTTGGCCCTTCTCAGTTCGCTA | 2374 | 2.67 |
| **9** | GGTCTTAGGAACTTTGCTGCCACAATA**--**TTGGCCCTTCTCAGTTCGCTA | 2166 | 2.44 |
| **10** | GGTCTTAGGAACTTTGCTGCCACAA**----**TTGGCCCTTCTCAGTTCGCTA | 1633 | 1.84 |
